# Automatic discrimination between safe and unsafe swallowing using a reputation-based classifier

**DOI:** 10.1186/1475-925X-10-100

**Published:** 2011-11-15

**Authors:** Mohammad S Nikjoo, Catriona M Steele, Ervin Sejdić, Tom Chau

**Affiliations:** 1 Bloorview Research Institute, Holland Bloorview Kids Rehabilitation Hospital, 150 Kilgour Road, Toronto, M4G 1R8, Canada and the Institute of Biomaterials and Biomedical Engineering, 164 College Street, Toronto, M5S 3G9, Canada and the Edward S. Rogers Sr. Dept. of Electrical and Computer Engineering, University of Toronto, 10 King's College Road, Toronto, M5S 3G4, Canada; 2 Toronto Rehabilitation Institute, 50 University Avenue, Toronto, M5G 2A2, Canada and the Department of Speech-Language Pathology, University of Toronto, 160-500 University Avenue, Toronto, M5G 1V7, Canada; 3 Department of Electrical and Computer Engineering, Swanson School of Engineering, University of Pittsburgh, 151 Benedum Hall, Pittsburgh, 15261, USA; 4 Institute of Biomaterials & Biomedical Engineering, University of Toronto, 164 College Street, Toronto, M5S 3G9, Canada and Bloorview Research Institute, Holland Bloorview Kids Rehabilitation Hospital, 150 Kilgour Road, Toronto, M4G 1R8, Canada

## Abstract

**Background:**

Swallowing accelerometry has been suggested as a potential non-invasive tool for bedside dysphagia screening. Various vibratory signal features and complementary measurement modalities have been put forth in the literature for the potential discrimination between safe and unsafe swallowing. To date, automatic classification of swallowing accelerometry has exclusively involved a single-axis of vibration although a second axis is known to contain additional information about the nature of the swallow. Furthermore, the only published attempt at automatic classification in adult patients has been based on a small sample of swallowing vibrations.

**Methods:**

In this paper, a large corpus of dual-axis accelerometric signals were collected from 30 older adults (aged 65.47 ± 13.4 years, 15 male) referred to videofluoroscopic examination on the suspicion of dysphagia. We invoked a reputation-based classifier combination to automatically categorize the dual-axis accelerometric signals into safe and unsafe swallows, as labeled via videofluoroscopic review. From these participants, a total of 224 swallowing samples were obtained, 164 of which were labeled as unsafe swallows (swallows where the bolus entered the airway) and 60 as safe swallows. Three separate support vector machine (SVM) classifiers and eight different features were selected for classification.

**Results:**

With selected time, frequency and information theoretic features, the reputation-based algorithm distinguished between safe and unsafe swallowing with promising accuracy (80.48 ± 5.0%), high sensitivity (97.1 ± 2%) and modest specificity (64 ± 8.8%). Interpretation of the most discriminatory features revealed that in general, unsafe swallows had lower mean vibration amplitude and faster autocorrelation decay, suggestive of decreased hyoid excursion and compromised coordination, respectively. Further, owing to its performance-based weighting of component classifiers, the static reputation-based algorithm outperformed the democratic majority voting algorithm on this clinical data set.

**Conclusion:**

Given its computational efficiency and high sensitivity, reputation-based classification of dual-axis accelerometry ought to be considered in future developments of a point-of-care swallow assessment where clinical informatics are desired.

## 1 Introduction

Dysphagia refers to any swallowing disorder [1] and may arise secondary to stroke, multiple sclerosis, and eosinophilic esophagitis, among many other conditions [2]. If unmanaged, dysphagia may lead to *aspiration pneumonia *in which food and liquid enter the airway and into lungs [3]. The video-fluoroscopic swallowing study (VFSS) is the gold standard method for dysphagia detection [4]. This method entails a lateral X-ray video recorded during ingestion of a barium-coated bolus. The health of a swallow is then judged by clinical experts according to criteria such as the depth of airway invasion and the degree of bolus clearance after the swallow. However, this technique requires expensive and specialized equipment, ionizing radiation and significant human resources, thereby precluding its use in the daily monitoring of dysphagia [5]. *Swallowing accelerometry *has been proposed as a potential adjunct to VFSS. In this method, the patient wears a dual-axis accelerometer infero-anterior to the thyroid notch. Swallowing events are automatically extracted from the recorded acceleration signals and pattern classification methods are then deployed to discriminate between healthy and unhealthy swallows. It is important to distinguish between swallowing vibrations and swallowing sounds, based on current evidence in the literature. Swallowing sounds have been largely attributed to pharyngeal reverberations arising from opening and closing of valves (oropharyngeal, laryngeal and esophageal valves), action of various pumps (pharyngeal, esophageal, and respiratory pumps) and vibrations of the vocal tract [6]. In contrast, in swallowing accelerometry, vocalizations are explicitly removed by preprocessing [7] and studies have implicated hyolaryngeal motion as the primary source of the acceleration signal [8,9]. Fundamentally, both the method of transduction and the primary physiological source of these signals are different. Our focus here is swallowing vibrations and recent progress in swallowing accelerometry is reviewed below.

### 1.1 Automatic classification

Das, Reddy & Narayanan [10] deployed a fuzzy logic-committee network to distinguish between swallows and 'artifacts' using time and frequency domain features of single-axis accelerometry signals. Although they achieved very high accuracies, their sample of swallows and 'artifacts' was very modest. Using a radial basis classifier with statistical and energetic features, Lee *et al. *[11] detected aspirations from single-axis cervical acceleration signals with approximately 80% sensitivity and specificity in a large pediatric cerebral palsy population. Both of these studies only examined accelerations in the anterior-posterior anatomical direction. However, recent research has shown that there is distinct information about swallowing that is encoded in the superior-inferior vibration [12]. Further, hyolaryngeal motion associated with swallowing is inherently two-dimensional and this motion was implicated as the likely source of swallow vibrations [9].

In the first dual-axis classification study, Lee *et al. *[5] discriminated between no airway invasion and airway invasion past the true vocal folds in 24 adult stroke patients using a variety of classifiers (linear discriminant, neural network, probabilistic network and nearest neighbor). A genetic algorithm (GA) selected the most discriminatory feature combinations. With linear classifiers, an adjusted accuracy of 74.7% was achieved in feature spaces of up to 12 dimensions.

In the aforementioned studies, various genres of features have demonstrated discriminatory potential. These include statistical features such as dispersion ratio and normality [11], time-frequency features such as wavelet energies [12], information theoretic features such as entropy rate [13], temporal features such signal memory [14], and spectral features such as the spectral centroid [15]. Further, there is evidence to suggest that complementary measurement modalities, such as nasal air flow and submental mechanomyography [16] may enhance segmentation and classification. Given the presence of multiple feature genres and different measurement modalities, the swallow detection and classification problem lends itself to a multi-classifier approach. For example, it may be sensible to dedicate one classifier to each feature genre [17].

In this paper, we invoke a novel, computationally efficient reputation-based classifier combination to automatically categorize dual-axis accelerometric signals from adult patients into safe and unsafe swallows, as labeled via videofluoroscopic review. We consider multiple feature genres from both anterior-posterior and superior-inferior axes and examine a much larger data set than that of previous swallow accelerometry classification studies.

## 2 Methods

### 2.1 Data collection

In this paper, we re-examine data from a subset of participants originally reported in [18]. Briefly, we recruited 30 patients (aged 65.47 ± 13.4 years, 15 male) with suspicion of neurogenic dysphagia who were referred to routine videofluoroscopic examination at one of two local hospitals. Patients had dysphagia secondary to stroke, acquired brain injury, neurodegenerative disease, and spinal cord injury. Research ethics approval was obtained from both participating hospitals.

**Figure 1:**
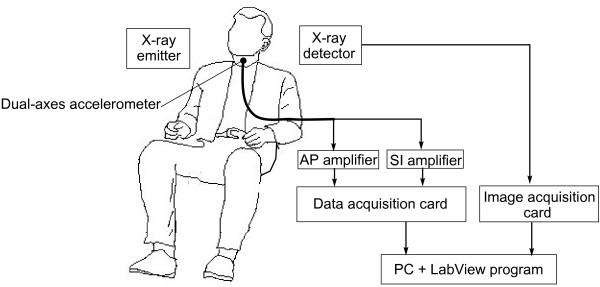
**Data Collection Set-up**.

The data collection set-up is shown in Figure 1.

Sagittal plane videofluoroscopic images of the cervical region were recorded to computer at a nominal 30 frames per second via an analog image acquisition card (PCI-1405, National Instruments). Each frame was marked with a timestamp via a software frame counter. A dual-axis accelerometer (ADXL322, Analog Devices) was taped to the participants neck at the level of the cricoid cartilage. The axes of the accelerometer were aligned to the anatomical anterior-posterior (AP) and superior-inferior (SI) axes. Signals from both the AP and SI axes were passed through separate pre-amplifiers each with an internal bandpass filter (Model P55, Grass Technologies). The cutoff frequencies of the bandpass filter were set at 0.1 Hz and 3 kHz. The amplifier gain was 10. The signals were then sampled at 10 kHz using a data acquisition card (USB NI-6210, National Instruments) and stored on a computer for subsequent analyses. A trigger was sent from a custom LabView virtual instrument to the image acquisition card to synchronize videofluoroscopic and accelerometric recordings. The above instrumentation settings replicate those of previous dual-axis swallowing accelerometry studies [7,9,13-15,19,20].

Each participant swallowed a minimum of two or a maximum of three 5 mL teaspoons of thin liquid barium (40%w/v suspension) while his/her head was in a neutral position. The number of sips that the participant performed was determined by the attending clinician. The recording of dual-axis accelerome-try terminated after the participant finished his/her swallows. However, the participant's speech-language pathologist continued the videofluoroscopy protocol as per usual. In total, we obtained 224 individual swallowing samples from the 30 participants, 164 of which were labeled as unsafe swallows (as defined below) and 60 as safe swallows.

### 2.2 Data segmentation

To segment the data for analysis, a speech-language pathologist reviewed the videofluoroscopy recordings. The beginning of a swallow was defined as the frame when the liquid bolus passed the point where the shadow of the mandible intersects the tongue base. The end of the swallow was identified as the frame when the hyoid bone returned to its rest position following bolus movement through the upper esophegeal sphincter. The beginning and end frames as defined above where marked within the video recording using a custom C++ program. The cropped video file was then exported together with the associated segments of dual-axes acceleration data. An unsafe swallow was defined as any swallow without airway clearance. Typically, this would include penetration and aspiration. Residue would be considered a situation of swallowing inefficiency that is not unsafe swallowing unless the residue was subsequently aspirated. Backflow is extremely rare in the oropharynx, and would only be classified as unsafe should it lead to penetration-aspiration. This definition of unsafe swallowing is in keeping with the industry standard Penetration-Aspiration Scale [21].

### 2.3 Pre-Processing

**Figure 2:**
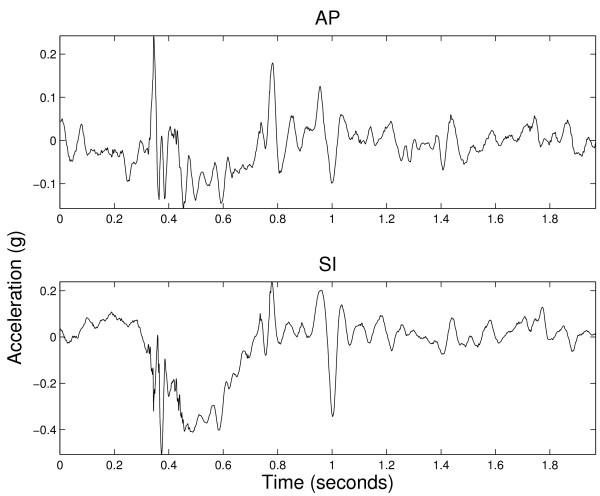
**Example of safe swallowing signals from AP and SI axes**.

**Figure 3:**
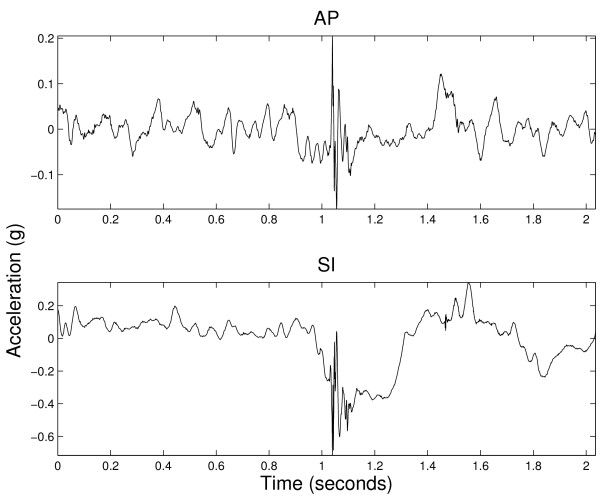
**Example of unsafe swallowing signals from AP and SI axes**.

It has been shown in [12] that the majority of signal power in swallowing vibrations of healthy adults lies below 100 Hz. However, given that we were dealing with patient data, we estimated the bandwidth of each of the 224 swallows as the spectral range from 0 Hz up to the frequency at which 95% of the signal energy was captured. We obtained average bandwidths of 175 ± 73 Hz and 226 ± 84 Hz for the AP and SI axes, respectively. Moreover, spectral centroids were < 70 Hz in both axes, suggesting that there is no appreciable signal energy beyond a few hundred Hz. Therefore, we downsampled all signals to 1 kHz. Vocalization was removed from each segmented swallow according to the normalized cross-correlation periodicity detector proposed in [7]. Whitening of the accelerometry signals to account for instrumentation nonlinearities was achieved using inverse filtering and autoregressive modeling [15]. Finally, the signals were denoised using a Daubechies-8 wavelet (8db) transform with soft thresholding. As detailed in [20], both the decomposition level and the wavelet coefficients were chosen to minimize the reconstruction error within a reduced wavelet subspace. Figures 2 and 3 exemplify pre-processed safe and unsafe swallowing signals, respectively.

### 2.4 Feature Extraction

Let *S *be a pre-processed acceleration time series, *S *= {*s*_2_, *s*_2_, ..., *s*_*n*_}. As in previous accelerometry studies, signal features from multiple domains were considered [13,16]. The different genres of features are summarized below.

1. Time Domain Features

• The sample mean is an unbiased estimation of the location of a signal's amplitude distribution and is given by,

(1) $$\mu_{s}=\frac{1}{n}\overset{n}{\underset{i=1}{\sum }}S_{i}.$$

• The variance of a distribution measures its spread around the mean and reflects the signal's power. The unbiased estimation of variance can be obtained as

(2) $$\sigma_{s}^{2}=\frac{1}{n-1}\overset{n}{\underset{i=1}{\sum }}{\left(S_{i}-\mu_{s}\right)}^{2}.$$

• The median is a robust location estimate of the amplitude distribution. For the sorted set *S*, the median can be calculated as

(3) $$MED\left(s\right)=\left\{\begin{array}{cc} S_{v}+1, & \text{if}\;n=2v+1;\\ \frac{s_{v}+s_{v+1}}{2}, & \text{if}\;n=2v. \end{array}\right)$$

• Skewness is a measure of the symmetry of a distribution. This feature can be computed as follows.

(4) $$\gamma_{1,s}=\frac{\frac{1}{n}\sum_{i=1}^{n}{\left(S_{i}-\mu_{s}\right)}^{3}}{{\left(\frac{1}{n}\sum_{i=1}^{n}{\left(S_{i}-\mu_{s}\right)}^{2}\right)}^{1.5}}.$$

• Kurtosis reflects the peakedness of a distribution. A high kurtosis value indicates a distribution with a sharp, narrow peak and heavy tails while a low kurtosis value signifies a distribution with a flattened peak and thin tails. This feature was computed as:

(5) $$\gamma_{2,s}=\frac{\frac{1}{n}\sum_{i=1}^{n}{\left(S_{i}-\mu_{s}\right)}^{4}}{{\left(\frac{1}{n}\sum_{i=1}^{n}{\left(S_{i}-\mu_{s}\right)}^{2}\right)}^{2}}.$$

2. Frequency Domain Features

• The peak magnitude value of the Fast Fourier Transform (FFT) of the signal *S *was also used as a feature. All the FFT coefficients were normalized by the length of the signal, *n*.

• The centroid frequency of the signal *S *[15] was estimated as

(6) $$\hat{f}=\frac{\int_{0}^{f_{max}}f|F_{s}\left(f\right){|}^{2}df}{\int_{0}^{f_{{}_{max}}}|F_{s}\left(f\right){|}^{2}df},$$

where *F*_*s*_(*f*) is the Fourier transform of the signal *S *and *f*_*max *_is the Nyquist frequency (effectively 500 Hz after downsampling).

• The bandwidth of the spectrum was computed using the following formula

(7) $$BW=\sqrt{\frac{{\int_{0}^{f_{max}}\left(f-\hat{f}\right)}^{2}|F_{s}\left(f\right){|}^{2}df}{\int_{0}^{f_{max}}|F_{s}\left(f\right){|}^{2}df}.}$$

3. Information Theory-Based Features

• The entropy rate [22] of a signal quantifies the extent of regularity in that signal. The measure is useful for signals with some relationship among consecutive signal points. We first normalized the signal *S *to zero-mean and unit variance. Then, we quantized the normalized signal into 10 equally spaced levels, represented by the integers 0 to 9, ranging from the minimum to maximum value. Now, the sequence of *U *consecutive points in the quantized signal, $Ŝ=\left\{{ŝ}_{1},{ŝ}_{2},...,{ŝ}_{3}\right\}$, was coded using the following equation

(8) $$a_{i}={ŝ}_{i+U-1}\cdot 10^{U-1}+...+{ŝ}_{i}\cdot 10^{0},$$

with *i *= 1, 2, ..., *n *- *U *+ 1. The coded integers comprised the coding set *A*_*U *_= {*a*_1_, ..., *a*_*n-U*+1_}. Using the Shannon entropy formula, we estimated entropy

(9) $$E\left(U\right)=-\overset{10^{U}-1}{\underset{t=0}{\sum }}P_{A_{U}}\left(t\right)\cdot \ln P_{A_{U}}\left(t\right),$$

where $p_{A_{U}}\left(t\right)$ represents the probability of observing the value *t *in *A*_*U*_, approximated by the corresponding sample frequency. Then, the entropy rate was normalized using the following equation

(10) $$\hat{E\left(U\right)}=\frac{E\left(U\right)-E\left(U-1\right)+E\left(1\right)\cdot \beta }{E\left(1\right)},$$

where $\hat{E\left(U\right)}$ denotes the normalized entropy, and *β *was the percentage of the coded integers in *A*_*L *_that occurred only once. Finally, the regularity index *ρ *∈ [0, 1] was obtained as

(11) $$\rho =1-\min\hat{E\left(U\right)},$$

where a value of *ρ *close to 0 signifies maximum randomness while *ρ *close to 1 indicates maximum regularity.

• To calculate the memory of the signal [13], its autocorrelation function was computed from zero to the maximum time lag (equal to the length of the signal) and normalized such that the autocorrelation at zero lag was unity. The memory was estimated as the time required for the the autocorrelation to decay to 1*/e *of its zero lag value.

• Lempel-Ziv (L-Z) complexity [23] measures the predictability of a signal. To compute the L-Z complexity for signal *S*, first, the minimum and the maximum values of signal points were calculated and then, the signal was quantized into 100 equally spaced levels between its minimum and maximum values. Then, the quantized signal, $B_{1}^{n}=\left\{b_{1},b_{2},...,b_{n}\right\}$, was decomposed into *T *different blocks, $B_{1}^{n}=\left\{\psi_{1},\psi_{2},...,\psi_{T}\right\}$. A block *ψ *was defined as

(12) $$\Psi =B_{j}^{\ell }=\left\{b_{j},b_{j+1},...,b_{\ell }\right\},1\leq j\leq \ell \leq n.$$

The values of the blocks can be calculated as follows:

(13) $$\Psi =\left\{\begin{array}{cc} \psi_{m}=b_{1}, & \text{if}\;\text{m}=1,\\ \psi_{m+1}=B_{h_{m}+1}^{h_{m+1}}, & m\geq 1, \end{array}\right)$$

where *h*_*m *_is the ending index for *ψ*_*m*_, such that *ψ*_*m*+1 _is a unique sequence of minimal length within the sequence $B_{1}^{h_{m+1}-1}$. Finally, the normalized L-Z complexity was calculated as

(14) $$LZ=\frac{T\log_{100}n}{n}.$$

### 2.5 Reputation-Based Classification

Reputation typically refers to the quality or integrity of an individual component within a system of interacting components. The notion of reputation has been widely used to ascertain the health of nodes in wireless networks [24], identify malicious hosts in a distributed system [25] and detect free-riders in peer-to-peer networks [26], among many other practical applications. Here, we apply the concept of reputation to judiciously combine decisions of multiple classifiers for the purpose of differentiating between safe and unsafe swallows. The general idea is to differentially weigh classifier decisions on the basis of their past performance.

The past performance of the *i*^*th *^classifier is captured via its *reputation*, $r_{i}\in ℜ,0\leq r_{i}\leq 1$, where 1 signifies a strong classifier (high accuracy) and 0 denotes a weak classifier. Briefly, the classifier is formulated as follows. Let Θ = {*θ*_1_, *θ*_2_,..., *θ*_*L*_} be a set of *L *≥ 2 classifiers and Ω = {*ω*_1_, *ω*_2_, ..., *ω*_*c*_} be a set of *c *≥ 2 class labels, where *ω*_*j *_≠ *ω*_*k*_, ∀*j *≠ *k*. Without loss of generality, Ω ⊂ ℕ. The input of each classifier is the feature vector $x\in R^{n_{i}}$, where *n*_*i *_is the dimension of the feature space for the *i*^*th *^classifier *θ*_*i*_, whose output is a class label *ω*_*j*_, *j *= 1, ..., *c*. Let *p*(*ω*_*j*_) be the *prior probability *of class *ω*_*j*_.

1. For a classification problem with *c *≥ 2 classes, we invoke *L *≥ 2 individual classifiers.

2. After training the *L *classifiers individually, the respective accuracy of each is evaluated using a validation set and expressed as a real number in [0, 1]. This number is the reputation of the classifier.

3. For each feature vector, *x*, in the test set, *L *decisions are obtained using the *L *distinct classifiers:

(15) $$\Omega \left(x\right)=\left\{\theta_{1}\left(x\right),\theta_{2}\left(x\right),...,\theta_{L}\left(x\right)\right\}.$$

4. We sort the reputation values of the classifiers in descending order,

(16) $$R^{*}=\left\{r_{1^{*}},r_{2^{*}},...,r_{L^{*}}\right\},$$

such that *r*_1* _≥ *r*_2* _≥ ··· ≥ *r*_L*_. Then, using this set, we rank the classifiers to obtain a reputation-ordered set of classifiers, Θ*.

(17) $$\Theta^{*}=\left(\begin{array}{c} \theta_{1^{*}}\\ \theta_{2^{*}}\\ \vdots \\ \theta_{L^{*}} \end{array}\right).$$

The first element of this set corresponds to the classifier with the highest reputation.

5. Next, we examine the votes of the first *m *elements of the reputation-ordered set of classifiers, with

(18) $$m=\left\{\begin{array}{cc} \;\;\frac{L}{2}, & \text{if}\;L\;\text{is}\;\text{even},\\ \frac{L+1}{2}, & \text{if}\;L\;\text{is}\;\text{odd}. \end{array}\right)$$

If the top *m *classifiers vote for the same class, *ω*_*j*_, we accept the majority vote and take *ω*_*j *_as the final decision of the system. However, if the votes of the first *m *classifiers are not equal, we consider the classifiers' individual reputations (Step 2) in arriving at the final decision, as detailed in step 6.

6. The probability that the combined classifier decision is *ω*_*j *_given the input vector *x *and the individual local classifier decisions is denoted as the *posterior probability*,

(19) $$p\left(w_{j}|\theta_{1}\left(x\right),\theta_{2}\left(x\right),...,\theta_{L}\left(x\right)\right)$$

which can be estimated using Bayes rule as

(20) $$p\left(w_{j}|\theta_{1},...,\theta_{L}\right)=\frac{\prod_{i=1}^{L}p\left(\theta_{i}|w_{j}\right)p\left(w_{j}\right)}{\sum_{t=1}^{c}\prod_{i=1}^{L}p\left(\theta_{i}|w_{t}\right)p\left(w_{t}\right)}.$$

when the classifiers are independent. For notational convenience, we have dropped the argument for *θ *above, but it is understood to be a function of *x*. The local likelihood functions, *p*(*θ*_*i*_|*ω*_*j*_), are estimated by the reputation values calculated in Step 2. When the correct class is *ω*_*j *_and classifier *θ*_*i *_classifies *x *into the class *ω*_*j*_, i.e., *θ*_*i*_(*x*) = *ω*_*j*_, we can write

(21) $$p\left(\theta_{i}=w_{j}|w_{j}\right)=r_{i}.$$

In other words, *p*(*θ*_*i *_= *ω*_*j*_|*ω*_*j*_) is the probability that the classifier *θ*_*i *_correctly classifies *x *into class *ω*_*j *_when *x *actually belongs to this class. This probability is exactly equal to the reputation of the classifier. On the other hand, when the classifier categorizes *x *incorrectly, i.e., *θ*_*i*_(*x*) ≠ *ω*_*j *_given that the correct class is *ω*_*j*_, then

(22) $$p\left(\theta_{i}\neq w_{j}|w_{j}\right)=1-r_{i}.$$

When there is no known priority among classes, we can assume equal prior probabilities. Hence,

(23) $$p\left(w_{1}\right)=p\left(w_{2}\right)=...=p\left(w_{c}\right)=\frac{1}{c}.$$

Thus, for each class, *ω*_*j*_, we can estimate the a posteriori probabilities as given by (20) using (21), (22), and (23). The class with the highest posterior probability is selected as the final decision of the system and the input subject *x *is categorized as belonging to this class.

### 2.6 Classifier evaluation

We ranked the signal features introduced above using the Fisher ratio [27] for univariate separability. In the time domain, mean and variance in the AP axis and skewness in the SI axis were the top-ranked features. Similarly, in the frequency domain, the peak magnitude of the FFT and the spectral centroid in the AP direction and the bandwidth in the SI direction were retained. Finally, in the information theoretic domain, entropy rate for the SI signal and memory of the AP signal were the highest ranking features. Subsequently, we only examined these feature subsets for classification, i.e., in total 8 different features were selected. For comparison between single and dual-axes classifiers, we also considered classifiers that employed feature subsets (as identified above) from a single axis.

Swallows from all 30 participants were pooled together. Given the disproportion of safe and unsafe samples, we invoked a smooth bootstrapping procedure [28] to balance the classes. All features were then standardized to zero mean and unit variance. Three separate support vector machine (SVM) classifiers [29] were invoked, one for each feature genre (time, frequency and information theoretic). Hence, the feature space dimensionalities for the classifiers were 3 (SVM with time features), 3 (SVM with frequency features) and 2 (SVM with information-theoretic features).

The use of different feature sets for each classifier increases the likelihood that the classifiers will perform independently [30].

Classifier accuracy was estimated via a 10-fold cross validation with a 90%-10% split. In each fold, performance on the training set was used to estimate the individual classifier reputations. Classifiers were then ranked according to their reputation values. Without loss of generality, assume *r*_1 _≥ *r*_2 _≥ *r*_3_. If *θ*_1 _and *θ*_2 _cast the same vote about a test swallow, their common decision was accepted as the final classification. However, if they voted differently, the *a posteriori *probability of each class was computed using (20) and the maximum *a posteriori *probability rule was applied to select the final classification.

## 3 Results

**Figure 4:**
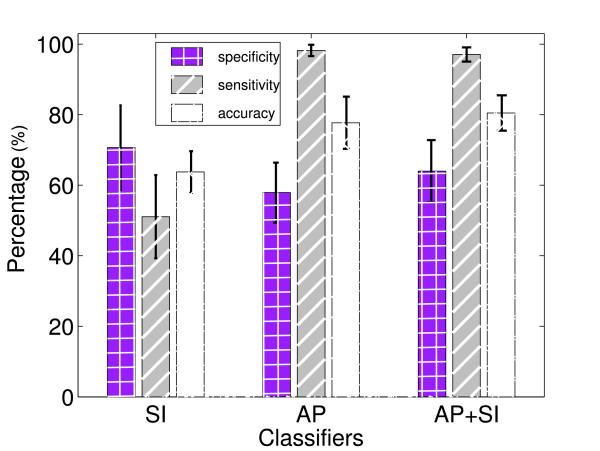
**Classification performance for the single-axis (AP, SI) and dual-axis (AP + SI) reputation-based classifiers**. The height of each bar denotes the average of the respective performance measure while the error bars denote standard deviation around the mean

The sensitivity, specificity and accuracy of the single-axis and dual-axis accelerometry classifiers are summarized in Figure 4. The dual-axis classifier had significantly higher accuracy (80.48 ± 5.0%) than either single-axis classifier (*p *<< 0.05, two-sample t-test), specificity (64 ± 8.8%) comparable to that of the SI classifier (*p *= 1.0) and sensitivity (97.1 ± 2%) on par with that of the AP classifier (*p *= 1.0). In other words, the dual-axis classifier retained the best sensitivity and specificity achievable with either single-axis classifier.

**Figure 5:**
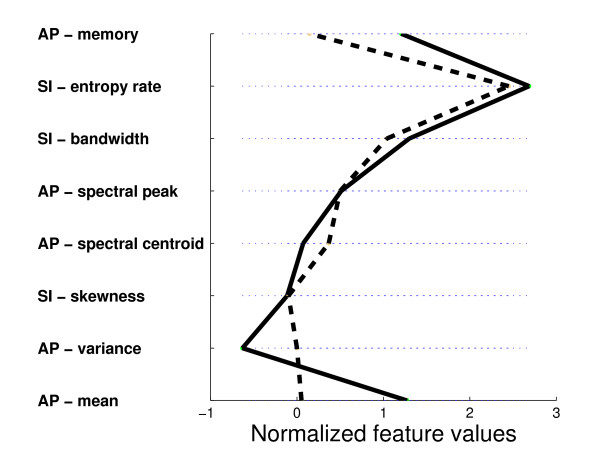
**Parallel axes plot depicting the internal representation of safe (solid line) and unsafe (dashed line) swallows**.

Figure 5 is a parallel axes plot depicting the internal representation of safe and unsafe swallows acquired by the reputation-based classifier. Each feature has been normalized by its standard deviation to facilitate visualization. On each axis, the median feature value is shown. The median values of adjacent axes are joined by solid (safe swallow) or dashed (unsafe swallow) lines.

## 4 Discussion

### 4.1 Dual versus single axis

Of the two axes, the AP axis tended to carry more useful information than the SI direction for discrimination between safe and unsafe swallowing. This observation is evidenced in Figure 4, where AP accuracy is dramatically higher than SI levels, echoing the findings of [12] who suggested that the AP axis is richer in information content (i.e., higher entropy) relating to swallowing. Note that data collection conditions and experimental protocols of the present study were similiar to that of [12]. Nonetheless, the SI axis does carry information distinct from that of the AP orientation, as dual-axis classification exceeds any single-axis counterpart. Our results thus support the inclusion of selected features from *both *the AP and SI axes for the automatic discrimination between safe and unsafe swallowing. Indeed, when comparing AP and SI signals, [12] reported minimal mutual information, and inter-axis dissimilarities in the scalograms, pseudo-spectra and temporal evolution of low- and high-frequency content.

In a recent videofluoroscopic study, both AP and SI accelerations were attributed to the planar motion of the hyoid and larynx during swallowing [9]. In that study, the displacement of the hyoid bone and larynx along with their interaction explained over 70% of the variance in the doubly integrated acceleration in both AP and SI axes at the level of the cricoid cartilage. This physiological basis of swallow accelerometry suggests that differences in hyolaryngeal motion between safe and unsafe swallowing are manifested in our selected features. Indeed, early single-axis accelerometry research had implicated decreased laryngeal elevation as the reason for suppressed AP accelerations in individuals with severe dysphagia [8].

### 4.2 Internal representation

In Figure 5, we immediately observe some distinct patterns which characterize each type of swallow. In the AP axis, unsafe swallows tend to have lower acceleration amplitude, higher variance, higher spectral centroid and shorter memory. The lower mean vibration amplitude in unsafe swallowing resonates with previous reports of suppressed peak acceleration [8] in dysphagic patients and reduced peak anterior hyoid excursion [31] in older adults, both suggesting compromised airway protection. The observation of a higher spectral centroid in unsafe swallowing may reflect departures from the typical axial high-low frequency coupling trends of normal swallowing as detailed in [12]. Likewise, the shorter memory and hence faster decay of the autocorrelation may be indicative of compromised overall coordination in unsafe swallowing.

It is also interesting to note that unsafe swallows tend to be negatively skewed while safe swallows are evenly split between positive and negative skew. In other words, in unsafe swallowing, the upward motion of the hyolaryngeal structure appears to have weaker accelerations than during the downward motion. This is opposite of the tendency reported in [12] for healthy swallowing and may reflect inadequate urgency to protect the airway.

### 4.3 Reputation-based classification

**Figure 6:**
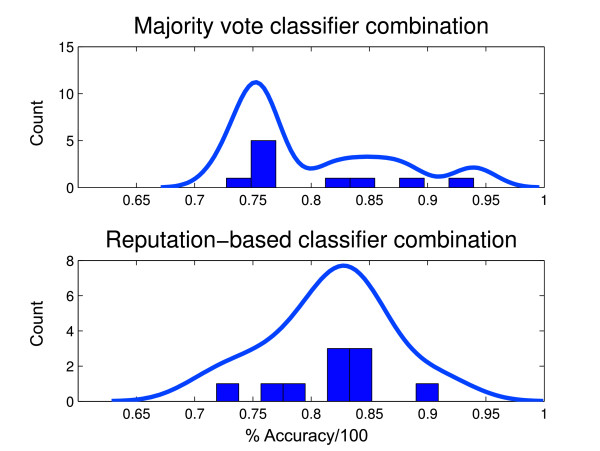
**Visual comparison of the densities of classification accuracies by majority voting (top) and reputation-based classification (bottom) for safe and unsafe swallow discrimination**.

The merit of a reputation-based classifier for the present problem can be appreciated by contrasting its performance against that of the classic method of combining classifiers, i.e., via the majority voting algorithm. To this end, Figure 6 summarizes the accuracies of both approaches from a 10-fold cross-validation using the data of this study. The accuracy, specificity, and sensitivity of classification using the majority voting algorithm on these data were 76.10%, 56.66%, and 94.51%, respectively. The histograms summarize the distribution of accuracies obtained from cross-validation. To aid in the visualization of underlying differences in performance, the corresponding density estimate (solid line) was obtained using a semi-parametric maximum likelihood estimator based on a finite mixture of Gaussian kernels. Clearly, the location of the density of reputation-based accuracies appears to be further to the right of the location of the majority voting density. The large spread in both densities amplifies the risk of Type II error and thus conventional testing (e.g., Wilcoxon ranksum) fails to identify any differences. However, upon more careful inspection using a two-sample Kolmogorov-Smirnoff test of the 20% one-sided trimmed densities (i.e., omitting the 2 most extreme points in each density), a statistically significant difference between the distributions (*p *= 0.0098) is confirmed.

The reputation-based classifier achieved higher adjusted accuracies (> 85%; average of sensitivity and specificity) than those reported in [5] (no greater than 75%). Patients were similarly aged and all had neurogenic dysphagia. Similar to the present study, the authors in [5] considered any entry into the airway as unsafe swaloowing. However, some key differences between the studies are worthy of mention. The present study had a slightly larger sample size, a better balance between males and females ([5] almost exclusively had males), and most importantly, a more significant representation of unsafe swallows (73% of total swallows compared to only 13% in [5]). Arguably, vibration patterns of pathological swallows vary more widely than those of safe swallows and hence a more comprehensive representation of the former may be well-justified.

Generally, the reputation-based classification scheme mitigates the risk of the overall classifier performance being unduly affected by a poorly performing component classifier within a multi-classifier system. Additionally, as exemplified in this study, the dimensionality of individual classifiers can be minimized, reducing the demand for voluminous training data.

### 4.4 Limitations

The dual-axes classifier attained very high sensitivity but modest specificity. In part, this bias towards higher sensitivity may be attributable to the preponderance of unsafe swallow examples in the original data set, despite our efforts to balance the classes via bootstrapping. In a practical system, it would mean that the classifier may overzealously flag a safe swallow as unsafe. This class imbalance issue may be a limitation of studying patients referred to videofluoroscopy, the majority of whom likely have a greater propensity for problematic swallowing. Hence, to obtain a larger number of safe swallows, a significantly expanded sample of patients may need to be recruited in the future.

The reputation classifier assumes independent features. This constrains the admissible features, but [12] has argued that many SI and AP features have low correlations. Future work may invoke independent component analysis or principal component analysis to generate additional novel independent features. The present classifier relies on static reputation values. In clinical application, the classifier may be trained and tested at different times with different patients. As a consequence, the feature distributions may change over time. In such case, dynamic reputation values may be more appropriate and future work may consider an online approach to dynamically update classifier reputations.

## 5 Conclusion

This study has demonstrated the potential for automatic discrimination between safe and unsafe (without airway clearance) swallows on the basis of a selected subset of time, frequency and information theoretic features derived from non-invasive, dual-axis accelerometric measurements at the level of the cricoid cartilage. Dual-axis classification was more accurate than single-axis classification. The reputation-based classifier internally represented unsafe swallows as those with lower mean acceleration, lower range of acceleration, higher spectral centroid, slower autocorrelation decay and weaker acceleration in the superior direction. Our results suggest that reputation-based classification of dual-axis swallowing accelerometry from adult stroke patients deserves further consideration as a clinical informatic in the management of swallowing disorders.

## Competing interests

The authors declare that they have no competing interests.

## Authors' contributions

MSN proposed and mathemathically formulated the static reputation-based algorithm, implemented the proposed algorithm and applied it to the problem of dysphagia detection, and wrote the entire manuscript. CS designed and oversaw the data collection protocol and critically reviewed the manuscript. ES helped in data collection, carried out swallow segmentation, and programmed some of the denoising methods. TC supervised this work and revised various versions of the manuscript. All authors read and approved the final manuscript.
